# Renal Cell Carcinoma With Myocardial Metastasis: A Case of Tumor-Induced Cardiac Tamponade After Radical Nephrectomy

**DOI:** 10.1155/crom/1493666

**Published:** 2025-09-23

**Authors:** Hussein Haidari, Samuel C. Hall, Sagar Kumar, Huseyin Kilic, Omar Alkharabsheh

**Affiliations:** ^1^Department of Internal Medicine, University of South Alabama, Mobile, Alabama, USA; ^2^Department of Medical Oncology, University of South Alabama, Mobile, Alabama, USA; ^3^Department of Pulmonary/Critical Care, University of South Alabama, Mobile, Alabama, USA; ^4^Department of Pathology, University of South Alabama, Mobile, Alabama, USA; ^5^Division of Medical Oncology, University of South Alabama, Mobile, Alabama, USA

**Keywords:** cardiac metastasis, cardiac tamponade, immunotherapy radical nephrectomy, renal cell carcinoma

## Abstract

Renal cell carcinoma (RCC) commonly metastasizes to the lung, bone, and liver. Cardiac metastasis, especially in the absence of IVC involvement, is considered extremely rare. We report an 81-year-old male with a history of clear cell RCC who underwent radical nephrectomy, and 10 years later, he presented with acute cardiac tamponade secondary to pathologically confirmed late myocardial metastasis.

## 1. Introduction

In the United States, renal cell carcinoma (RCC) accounts for 2%–3% of all adult malignant neoplasms and is considered the most lethal urologic cancer. Clear RCC is the most common histology, accounting for approximately 70%–80% of all renal malignancies [1]. RCC primarily affects the elderly population, and it is notorious for its mechanism of late metastasis to unusual anatomical sites. Late recurrences are common following curative treatment, such as radical nephrectomy. Cardiac involvement in RCC is rare and typically arises from direct tumor thrombus extension into the inferior vena cava [2]. Cardiac metastasis in the absence of vena cava extension is exceedingly rare, with only a few cases reported in the literature [3]. Here, we describe a case of late myocardial metastases from RCC in the absence of vena cava involvement. This case illustrates the importance of long-term surveillance in RCC patients and the potential for atypical metastatic presentations, necessitating a high index of suspicion for timely diagnosis and management.

## 2. Case Presentation

This is an 81-year-old male with a notable medical history of hypertension, diabetes mellitus, and prior RCC. He underwent right radical nephrectomy 10 years prior to his presentation, and his stage was pT1b N0 M0. During the surveillance period of 5 years, there was no evidence of recurrence of cancer. He reports sudden onset chest pain. Imaging and echocardiogram demonstrated a large pericardial effusion with tamponade that required an emergent pericardial window. During the procedure, hemorrhagic fluid was drained with ligation of the bleeding point at the lateral ventricle wall, resulting in immediate hemodynamic relief.

Further evaluation of the mass was completed via MRI of the chest, which showed a well-circumscribed tumor arising from the anterior ventricular wall. Given the history of RCC in the patient, cardiac metastases were a concern. The tumor was resected by cardiothoracic surgery.

**Figure 1:**
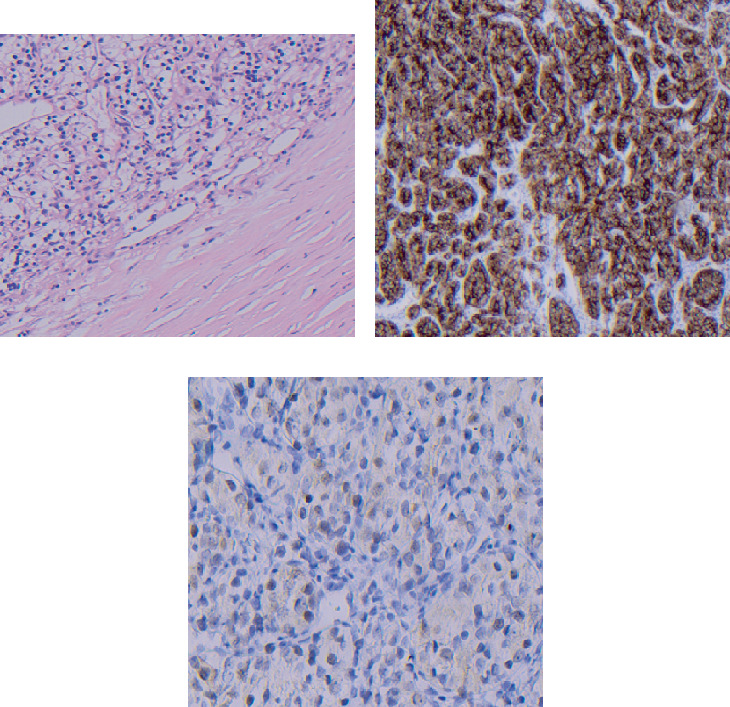
Histopathological overview of renal cell carcinoma metastasis to the heart. (a) On H&E, the slide shows a tumor composed of nests and sheets of cells with abundant clear cytoplasm, separated by a delicate thin-walled capillaries. (b) Diffuse membranous positivity for CD10 highlights the renal origin. (c) Nuclear positivity for PAX8 (+) further confirms renal epithelial differentiation.

The ventricular tumor was excised without complications. Pathology demonstrated malignant cells consistent with metastatic RCC of clear cell subtype (Figure 1a). Immunohistochemical stains showed strong positivity for CD10 (++) and PAX8 (+), markers commonly associated with RCC. This thus confirmed the diagnosis of RCC metastasis to the heart (Figure 1b,c).

After metastectomy, the patient had an unremarkable postoperative course and was discharged in stable condition. Initially, a CT scan of the chest, abdomen, and pelvis, including a bone scan, was negative; however, an FDG-PET was obtained to evaluate for residual metabolic disease and revealed minimal residual metabolic activity within the pericardium, likely related to an incomplete resection or further metastasis without other sites of disease. Confirmation came via circulating tumor DNA testing, which was positive on the Signatera assay secondary to the presence of tumor-specific DNA.

Multidisciplinary oncology team decided to start systemic immunotherapy with the programmed death ligand-1 inhibitor pembrolizumab.

## 3. Discussion

RCC often has a great predisposition toward metastasis, where about 20%–30% of the patients are usually diagnosed with either metastatic or locally advanced diseases upon their first presentation [3]. Cardiac metastasis is more common than primary cardiac tumors, with a secondary-to-primary ratio of 20:1. Ninety percent of primary cardiac tumors are benign. Cardiac metastasis can occur through various mechanisms, including dissemination via the bloodstream, direct extension from the mediastinum, or tumor invasion into the vena cava with subsequent growth into the right atrium. Lymphatic dissemination typically leads to pericardial metastases, while hematogenous spread is more likely to result in myocardial metastases [4].

The development of hemorrhagic pericardial effusion in cardiac metastasis is mainly attributed to the invasion of the pericardium by metastatic tumor cells. This invasion may occur via various routes, such as lymphatic spread, hematogenous dissemination, direct extension from nearby structures, or transvenous infiltration [5, 6].

A cardiac mass necessitates a thorough differential diagnosis to distinguish between tumor, vegetation, calcification, and thrombus potential etiologies. Cardiac masses can be detected through multimodal noninvasive imaging techniques.

Transthoracic echocardiography remains the primary diagnostic approach, enabling the assessment of the mass's size, contours, mobility, site of attachment, and hemodynamic effects. Contrast-enhanced TTE helps evaluate the perfusion of the mass, which assists in differentiating vascular tumors from thrombi. Cardiac magnetic resonance imaging is the most comprehensive noninvasive diagnostic tool, providing valuable information about the mass's topographic relationships, extension to surrounding structures, tissue characteristics, and specific contrast enhancement patterns. Histopathological examination remains the definitive diagnostic approach for any surgically removed cardiac tumor [4]. For metastatic RCC, immune checkpoint inhibitors and tyrosine kinase inhibitors are the standard of care. If resectable, metastasectomy followed by 1 year of pembrolizumab is the current recommendation based on the results of KEYNOTE-564 [7].

## 4. Conclusion

This is a case of cardiac metastasis in the absence of inferior vena cava involvement, which is a very rare manifestation of RCC. This case illustrates the importance of long-term surveillance in RCC patients and the potential for atypical metastatic presentations, necessitating a high index of suspicion for timely diagnosis and management.

## Data Availability

The data that support the findings of this study are available from the corresponding author upon reasonable request.
